# The effectiveness and safety of palbociclib and ribociclib in stage IV HR+/HER-2 negative breast cancer: a nationwide real world comparative retrospective cohort study

**DOI:** 10.3389/fonc.2023.1203684

**Published:** 2023-12-15

**Authors:** Nour Hisham Al-Ziftawi, Shereen Elazzazy, Mohammed Fasihul Alam, Asrul Shafie, Anas Hamad, Salha Bbujassoum, Mohamed Izham Mohamed Ibrahim

**Affiliations:** ^1^ Pharmacy Department, Aman Hospital, Doha, Qatar; ^2^ Pharmacy Department, The National Center for Cancer Care and Research, Hamad Medical Corporation, Doha, Qatar; ^3^ Department of Public Health, College of Health Sciences, QU Health, Qatar University, Doha, Qatar; ^4^ School of Pharmaceutical Sciences, Universiti Sains Malaysia, Gelugor, Malaysia; ^5^ College of Pharmacy, QU Health, Qatar University, Doha, Qatar; ^6^ Medical Department, The National Center for Cancer Care and Research, Hamad Medical Corporation, Doha, Qatar

**Keywords:** cyclin-dependent-kinase 4/6 inhibitors, HR+/HER-2 negative, advanced breast cancer (ABC), effectiveness & efficiency (E&E), CDK4/6 cell cycle inhibitors

## Abstract

**Introduction:**

Palbociclib and ribociclib are indicated in the first-line treatment of hormonal receptor-positive HER-2 negative (HR+/HER2- negative) advanced breast cancer. Although randomized-controlled trials (RCTs) proved their clinical efficacy, there are no observational studies yet to validate the clinical findings in the real-world. Therefore, this study aimed to evaluate and compare the clinical effectiveness and safety profiles of palbociclib and ribociclib in Qatar.

**Materials and methods:**

A retrospective observational study was conducted on HR+/HER-2-negative stage-IV breast cancer patients receiving palbociclib or ribociclib in the state of Qatar. Clinical data were collected from the National Center for Cancer Care and Research (NCCCR) in Qatar using Cerner^®^. Primary outcomes were progression-free-survival (PFS) and overall-survival (OS) generated by Kaplan-Meier curves. Moreover, safety profiles of both two treatments were evaluated.

**Results:**

The data from 108 patients were included in the final analysis. There was no statistically significant difference in PFS between the palbociclib and ribociclib groups; PFS was 17.85 versus 13.55 months, respectively(p> 0.05). Similarly, there was no statistically significant difference in OS between the two medications, 29.82 versus 31.72 months, respectively(p>0.05). Adverse events were similar between the two groups. Neutropenia was the most common side effect in the study population accounting for 59.3% of the patients.

**Conclusions:**

Therefore, both treatments have similar efficacy and safety profiles. Further research on a larger-scale population and longer follow-up period is recommeneded.

## 1 Introduction

Breast cancer is a prevalent non-communicable disease globally, with the highest incidence rate among all cancers (1). In 2018, it ranked first as the most commonly diagnosed cancer and lung cancer, accounting for around 2.1 million cases, representing 11.6% of the global cancer incidence burden, along with lung cancer (2). In addition, breast cancer was the leading cause of cancer-related mortality in females, accounting for 627,000 deaths (6.2% of the total cancer-related deaths and 15% of women’s cancer-related deaths) in 2018 (2, 3) Breast cancer treatment is complex and most probably requires a combination of different treatment modalities (4, 5). The treatment mainly relies on the classification of breast cancer, which can mainly be classified by stage as follows: early-stage breast cancer (stage I and II A), advanced breast cancer (stage IIB and stage III), and metastatic breast cancer (stage IV) (6, 7). Surgery is considered the mainstay treatment for nonmetastatic breast cancer, unless otherwise contraindicated, in combination with systematic therapy (chemotherapy, hormonal therapy, targeted biological therapy, and immune therapy), radiotherapy, or both. However, the treatment of metastatic breast cancer is usually based on systematic therapy (4).

Metastatic breast cancer, in specific, remains challenging due to being uncurable. The primary goals of treatment is to prolong the survival of patients while reducing treatment-related adverse events and toxicities to delay disease progression whilist maintaining or improving quality of life (8). This is because it has a low survival rate; the five-year survival rate of metastatic breast cancer is approximately 28% (9). The prognosis of patients with stage IV breast cancer can be different depending on the molecular subtype of the disease (hormone receptor (HR) and human epidermal growth factor receptor 2 (HER2) status), site(s) of metastasis, the number of sites involved in metastasis, the status of the lymph nodes, the previous treatment received, and the pathological and clinical characteristics of the disease (10, 11). Most breast cancer patients, including advanced and metastatic breast cancer patients, fall under the category of HR+/HER-2 negative molecular subtype (12). For instance, HR+/HER-2 negative accounted for 69% of the total breast cancer cases based on the 2016 to 2020 cases in the United States (13). Similar ratios were obtained from other countries as well (14–16). Therefore, for those HR+/HER-2 negative metastatic breast cancer patients, systematic drug therapy also remains the mainstay treatment. The first-line treatment for this category of patients, postmenopausal and premenopausal patients, is to receive hormonal therapy in combination with targeted cyclin-dependent kinase 4 and 6 inhibitors (CDK4/6 inhibitors), in the absence of a visceral crisis (17).

CDK4/6 inhibitors is a class of medications that target cyclin-dependent kinase 4, and 6 enzymes, which are important in the tumor cell cycle, inhibiting them leads to cell viability (18). Three agents have been approved in the first-line treatment for stage IV breast cancer under this class of medications: palbociclib, ribociclib, and abemaciclib (19). The three CDK4/6 inhibiting agents showed more favorable outcomes when combined with the mainstay hormonal therapy for HR+/HER-2 breast cancer patients in terms of prolonged survival compared to their comparator endocrine therapy alone (20–27). That is, palbociclib prolonged the progression-free survival (PFS) in advanced HR+/HER-2 negative breast cancer postmenopausal females when comparing palbociclib plus letrozole to letrozole monotherapy according to PALOMA-2 trial (23) and so did it as per the PALOMA-3 trial when comparing palbociclib plus fulvestrant to fulvestrant monotherapy (24). As for ribociclib, according to the MONALEESA-2 and MONALEES-3 trials, ribociclib demonstrated a significant improvement in the PFS compared to the letrozole and fulvestrant monotherapies, respectively, in advanced HR+/HER-2 negative breast cancer postmenopausal females (20, 21). In addition, in the MONALEESA-7 trial, ribociclib improved the PFS in premenopausal advanced HR+/HER-2 negative breast cancer women. Similarly, for the third CDK4/6 inhibiting agent, abemaciclib, phase III clinical trials proved the increase in the PFS when using abemaciclib in the progressive and in the first-line treatment of advanced HR+/HER-2 negative breast cancer postmenopausal females (25, 26). However, abemaciclib slightly differs from the other CDK4/6 inhibitors in that it was the only approved CDK4/6 inhibiting drug as monotherapy without endocrine therapy for HR+/HER-2 negative metastatic breast cancer without endocrine therapy (28, 29). Noteworthily, Only ribociclib and abemaciclib demonstrated a statistically significant increase in overall survival (OS) compared to the endocrine monotherapy (30–32). However, the efficacy of palbociclib in improving OS remains uncertain. Manufacturer-conducted landmark trials indicate that palbociclib, when combined with letrozole or fulvestrant, did not show a statistically significant difference in OS compared to standard therapy (33, 34). Nonetheless, some real-world evidence (RWE) from the US suggests a statistically significant improvement in OS for patients treated with palbociclib plus aromatase inhibitor (AI) compared to those receiving AI monotherapy (35).

Although these agents demonstrated clinical benefits, they can be associated with severe multiple blood side effects, cardiac arrhythmias, and many other side effects that can lead to toxicities, so regular monitoring is always required (19). To date, there is a limited number of observational studies based on RWE regarding the medications in this class. In addition, there is no head-to-head comparison between these medications in the class. Therefore, to fill this gap of knowledge, this study aims to investigate the effectiveness and safety of two CDK4/6 inhibitors, palbociclib and ribociclib, for real-world breast cancer patients’ data from the clinical practice in the state of Qatar.

## 2 Materials and methods

### 2.1 Settings

In this retrospective observational cohort study, real world data were collected from the computer-based medical records system (Cerner ^®^) including all Stage IV HR+/HER-2 negative Breast Cancer in Qatar, treated in the only cancer center in Qatar – the National Center for Cancer Care and Research (NCCCR). Prior to the actual start of this study, the study was firstly ethically approved by the Medical Research Center (MRC) on January 30, 2020, under the protocol approval number: MRC- 01-19-318, followed by the approval from the Qatar University International Review Board (QU-IRB) on February 10, 2020, under the approval number: QU-IRB- 1231- E/20.

### 2.2 Population and sample

Due to the retrospective nature of this study, the sampling method was sampling by convenience where all the medical records for patients who received either palbociclib or ribociclib in the specified data collection period as per the ethical approval, from January 2017 to December 2019, were included when eligible to ensure the reliability of the data by targeting the maximum sample size possible. Patients’ eligibility in the study was determined according to the following predetermined inclusion and exclusion criteria:

Inclusion criteria:

• Being a female breast cancer patient with stage IV breast cancer.
• Being hormonal -positive for either estrogen and progesterone (ER+ and PR+), or hormone receptor-positive for only estrogen receptors (ER+, PR negative).
• Having HER-2 negative cancerous cells as determined by immunohistochemistry (IHC). An IHC result of 0 to +1 means a weak representation of HER-2, whereas a score of 2 means a borderline and a score of +3 means an overexpression of HER-2 (36). Only IHC result of 0 to +1 are considered as negative expressions and are included in this study.
• Receiving appropriate combination with the treatments of comparison as approved by the FDA; i.e.: receiving palbociclib with either AI (anastrozole or letrozole) or fulvestrant or receiving ribociclib with either AI or fulvestrant or tamoxifen.
• According to the FDA, having a corresponding menopausal status to the treatment of interest. i.e., being ONLY postmenopausal while firstly receiving palbociclib with its selected combination or being premenopausal/perimenopausal/or postmenopausal when receiving ribociclib with its selected combination.
• Completing at least three cycles of palbociclib or ribociclib with their combinations.

Exclusion criteria:

• Male breast cancer patients.
• The cancer hormonal receptor status do not correspond to the ones included in the inclusion criteria, i.e., triple positive, triple-negative, or PR+ and ER-negative breast cancer.
• A cancer HER-2 protein expression status that does not match the HER-2 negative status; i.e.: week HER-2 expression (IHC result of +2), or a positive HER-2 expression (IHC result of +3).
• Receiving a non-FDA indicated combination with the treatment of interest (e.g., receiving tamoxifen alongside palbociclib).
• Receiving treatment with a non-corresponding menopausal status, i.e.: receiving palbociclib while still be premenopausal or perimenopausal.
• Completing less than three cycles of either of the two CDK4/6 inhibiting agents.
• Receiving one of the CDK4/6 inhibitors as a second-line after developing a disease progression on another CDK4/6 inhibitor, e.g., receiving a palbociclib as a second-line treatment after a patient developed a disease progression using a ribociclib.

### 2.3 Outcome measures

(i) Primary outcome measures

• Overall survival (OS) duration in months: it is the time in months that a patient lived for from the point of receiving one of the two treatments (palbociclib or ribociclib) till death, due to a progressed disease, side effect, hospitalization, or any other cause of death.
• Progression-free survival (PFS) duration in months: it is the time in months that a patient survives without developing a further progression or of her cancer condition (37).
• Death: it is the end of life of a patient either due to treatment side effects, new progression, or any other cause of death.

(ii) Secondary outcome measure

• Adverse drug reactions (ADRs). This included blood-related adverse drug reactions such as neutropenia and febrile neutropenia, anemia, thrombocytopenia, and pancytopenia. In addition, they include gastric-related side effects such as diarrhea, constipation, nausea and vomiting, and abdominal pain; cardiac side effects such as corrected QT interval prolongation (QTc prolongation); neuropathy and fatigue, and impaired liver functions.

### 2.4 Data collection and handling

Data collection was based on a predetermined data collection tool. The major parameters collected were: patient characteristics, menopausal status, breast cancer molecular subtype, whether a patient’s diagnosis of metastatic breast cancer was *de novo* or recurrent, recipient of previous hormonal therapy status, the name of the CDK4/6 inhibitor used for a patient with the dose and the combination, starting date, treatment discontinuation date (if applicable), disease progression date (if applicable). Additionally, the number of corresponding lab tests were collected before and after progression (if any). These included the number of complete blood count (CBC) lab tests, number of comprehensive metabolic panel (CMP) lab tests, number of liver function tests, number of endocrinology-related lab tests (e.g., vitamin D, vitamin B, TSH, and FSH levels), number of tumor markers and catecholamine tests, number of coagulation lab tests (PT, PTT, INR). Moreover, the corresponding clinical imaging and their counts for both before and after progression (if applicable) were collected. The clinical imaging of interest was: magnetic resonance imaging (MRI), computerized tomography (CT) scan, x-ray, ultrasound, mammogram, and the dual-energy X-ray absorptiometry (DXA) for bones. Besides, the number of cardiac electrocardiograms (ECG or EKG) records and echocardiogram scans were documented due to the reported possible cardiac side effects. Lastly, the date of death of the patient (if applicable) was documented.

Data was collected retrospectively for a 3-year period by one of the research team members in accordance with the pre-developed data collection sheet that was approved by the clinical data management team from the MRC at the study protocol development phase. The process of the data collection was overseen by the corresponding author, and the data was validated by the oncologist in the research team. The process of data validation used to take place frequently on a bi-monthly interval. In case of discrepancies, they are either brought to the investigator’s attention for clarification or resolved in-house through self-evident corrections among the remaining clinical research team members. Confidential information of patients such as name and date of birth was not collected, and each patient was given a unique code instead for future reference and remained anonymous. All data was handled confidentially by only the research team members.

### 2.5 Statistical analysis

Descriptive statistics were used to describe the main patients’ demographic characteristics, including nationality, age, menopausal status, hormonal receptors and HER-2 status, metastasis diagnosis status, and prior receive of hormonal therapy status. In addition, descriptive statistics were used to summarize the number of cycles completed in the two treatment groups (palbociclib and ribociclib), the number of patients who experienced side effects, and the overall hospitalization.

In correspondence to the primary outcomes, two time-to-event survival analyses using the Kaplan-Meier estimate were used; one was for the OS, and the other was for the PFS. Data were classified according to three major categories: time of the total follow-up, outcome (developing the event or censored, i.e., did not develop the event of interest during the following period), and treatment group (palbociclib or ribociclib groups). For the OS Kaplan-Meier estimate, the event of interest was ‘death’. Whereas, for the PFS Kaplan-Meier estimate, the event of interest was developing a new disease progression. For both Kaplan-Meier analyses, the follow-up time is the duration of months starting from the date a patient received either palbociclib or ribociclib to the date a patient developed the event of interest or the end of the follow-up period. The survival distribution for the two treatment groups for both curves was compared using the log-rank test at a significance level of 0.05.

A COX regression analysis was performed to explore the factors affecting the OS and the PFS. The independent variables entered into the COX-regression analysis were nationality, menopausal status, recipient of previous hormonal therapy, diagnosis of metastasis (*de novo* or recurrent), site of metastasis, and the combination medication(s) with the CDK4/6 inhibitors. All the statistical analyses considered results at a 5% level of significance and were conducted using the Statistical Package for the Social Sciences (SPSS)^®^ version 26 (38).

## 3 Results

A total number of 145 potentially eligible patients’ records were identified for screening during the period from 01.01.2017 to 31.12.2019. Out of the 145 total retrieved records, 37 records were excluded for not meeting the inclusion/exclusion criteria as follows: 12 records were excluded based on the menopausal status, five were excluded due to having different sub-molecular types based on the receptors and proteins status, i.e., being triple-positive breast cancer (n=3), or being triple-negative breast cancer (n=1), or being positive for the PR and not the ER (n=1), two more medical records were excluded for not being on an approved FDA combination with the indicated CDK4/6 inhibiting agent, and 18 were excluded for not completing at least three-cycles of the CDK4/6 inhibitor. Therefore, the total final eligible patients’ records were 108 based on exclusive 108 patients. As per the study inclusion/exclusion criteria, all the included records were based on female patients. The mean (SD) age of the population was 55.92± 10.59 years, with a median (IQR) of 55.00 (16) years. The population were from different races as follows: Arabs (n= 80; 74.1%), Asians (n= 13; 12%), Europeans (n= 11; 10.19%), South Africans (n=2; 1.9%), and South Americans (n=2; 1.9%). Most of the patients were ER+ PR+ HER-2-negative (77.8%), whereas the rest were ER+ PR-negative HER-2-negative. As for the diagnosis of metastasis, 63.9% of the patients received their diagnosis as a recurrent or progressive disease, whereas the rest of the population had it ‘de Novo. Bone was the most common site for metastasis, accounting for 36.1% of all the cases. Most of the patients received hormonal therapy before their first receiving of the CDK4/6 inhibitor drug, with 55.6% in the adjuvant setting and 25.9% in the metastatic setting. Most of those who received hormonal therapy in the adjuvant settings were resistant to hormonal therapy, meaning that they developed recurrence/metastasis while taking the hormonal therapy without completing the indicated period (26.9%). CDK4/6 inhibitor was the first line in metastasis for 43.5% of the population, whereas it was not the first line for 56.5%. Letrozole was the most common combination in the first-line treatment, with either palbociclib or ribociclib accounting for 56.5% among the patients, followed by fulvestrant, which accounted for 39.8% of the combinations among the population. The median number of cycles completed by patients on the CDK4/6 inhibiting agent was eight cycles. The population’s baseline characteristics are summarized in **Table 1**.

**Table 1:** Baseline Characteristic of Patients Receiving CDK4/6 Inhibitors.

	All Population (N=108)	Palbociclib Group (N=81)	Ribociclib Group (N=27)	P- Value
**Age, mean (SD)**	55.9 (10.6)	57.5 (10.5)	51.1 (9.5)	0.004*
**Nationality, n (%)**	34 (31.5)	28 (34.6)	6 (22.2)	0.618
Qatari	10 (9.3)	7 (8.6)	3 (11.1)	
Egyptian	9 (8.3)	7 (8.6)	2 (7.4)	
Sudanese	8 (7.4)	3 (3.7)	5 (18.5)	
Syrian	5 (4.6)	4 (4.9)	1 (3.7)	
Jordanian	14 (13.0)	13 (16.1)	1 (3.7)	
Other Arab nationals	11 (10.2)	9 (11.1)	2 (7.4)	
European	8 (7.4)	5 (6.2)	3 (11.1)	
Philippino	4 (3.7)	3 (3.7)	1 (3.7)	
Indian	1 (0.9)	0 (0.0)	1 (3.7)	
Bengali	2 (1.8)	1 (1.2)	1 (3.7)	
South African	2 (1.8)	1 (1.2)	1 (3.7)	
Latin America nationals				
**Measurements, median (IQR)**				0.059
Hight (cm)	156.70	156.50	158.00	0.796
	(8.00)	(8.65)	(11.80)
Weight (Kg)	74.70	75.00	74.00	0.324
	(19.45)	(20.60)	(17.20)
BMI (Kg/m^2^)	29.46	29.92	28.77	0.941
	(8.14)	(8.55)	(6.05)
BSA (m^2^)	1.81	1.81	1.81	
	(0.25)	(0.27)	(0.25)	
**Menopause Status, n (%)**				<0.001*
Pre-menopause	14 (13.0)	0 (0.0)	14 (51.9)	
Perimenopause	5 (4.6)	0 (0.0)	5 (18.5)	
Post-menopause	89 (82.4)	81 (100)	8 (29.6)	
**Breast Cancer Molecular Type, n (%)**				0.595
ER+ PR+ HER-2 –	84 (77.8)	62 (76.2)	22 (81.5)	
ER+ PR – HER-2 –	24 (22.2)	19 (23.5)	5 (18.5)	
**Metastatic Diagnosis, n (%)**				0.565
*De novo*	39 (36.1)	28 (34.6)	11 (40.7)	
Progressive	69 (63.9)	53 (65.4)	16 (59.3)	
**Metastasis Site, n (%)**				0.965
Lymph nodes only	17 (15.7)	13 (16.0)	4 (14.8)	
Bones ± lymph nodes	42 (38.9)	32 (39.5)	10 (37.0)	
Lungs with no liver	10 (9.3)	6 (7.4)	4 (14.8)	
Liver	14 (13.0)	10 (12.3)	4 (14.8)	
Other viscera	5 (4.6)	5 (6.2)	0 (0.0)	
Bones and viscera	20 (18.5)	15 (18.5)	5 (18.5)	
**Receiving Prior HRT, n (%)**				0.328
Yes	87 (80.6)	67 (82.7)	20 (74.1)	
No	21 (19.4)	14 (17.3)	7 (25.9)	
			
**Settings of Prior HRT, n (%)**				0.716
Adjuvant	60 (55.6)	45 (55.6)	15 (55.6)	0.23
Recurrent on HRT	29 (26.9)	18 (40)	11 (40.7)	
Recurrence <1 year after completion HRT	9 (8.3)	7 (15.5)	2 (7.4)	
Recurrence >1 year after completion of HRT	21 (19.4)	19 (42.2)	2 (7.4)	
Metastatic	28 (25.9)	22 (27.2)	6 (22.2)	0.175
Received 1 line HRT prior to CDK4/6 inhibitor	17 (15.7)	15 (18.5)	2 (7.4)	
Received 2 lines HRT prior to CDK4/6 inhibitor	9 (8.3)	5 (6.2)	4 (14.8)	
Received >2 lines HRT prior to CDK4/6 inhibitor	2 (1.8)	2 (2.4)	0 (0.0)	
**CDK4/6 Inhibitor was the first line, n (%)**				0.315
Yes	47 (43.5)	33 (40.7)	14 (51.9)	
No	61 (56.5)	48 (59.3)	13 (48.1)	
**CDK 4/6 Inhibitor Combination, n (%)**				0.512
AI (Anstrazole)	1 (0.9)	1 (1.2)	0 (0.0)	
AI (Letrozole)	56 (51.9)	40 (49.4)	16 (59.3)	
Fluvestrant	43 (39.8)	34 (42.0)	9 (33.3)	
Shifting between AI and Fluvestrant	7 (6.5)	6 (7.4)	1 (3.7)	
Tamoxifen	1 (0.9)	0 (0.0)	1 (3.7)	
**Number of CDK4/6 Inhibitor Cycles Completed,**				0.006*
**median (IQR)**	8 (8)	9 (9)	6 (4)	

**Figure 1:**
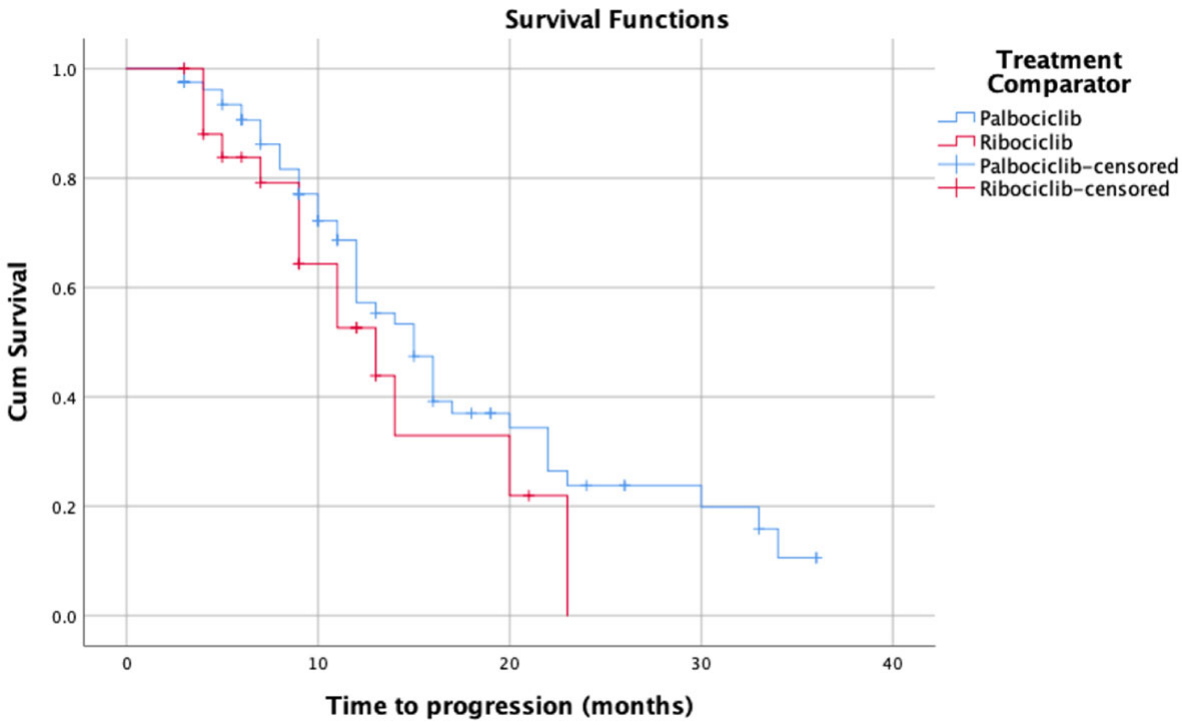
Kaplan-Meier’s survival curves of the progression-free survival for palbociclib and ribociclib.

**Figure 2:**
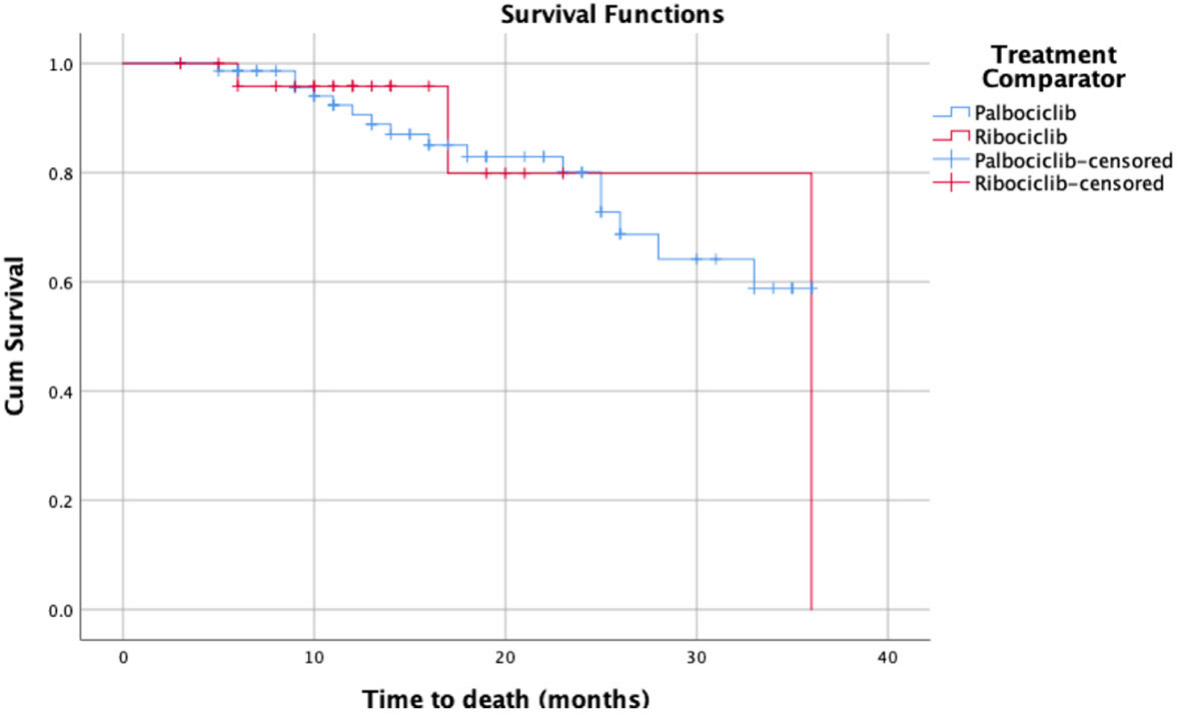
Kaplan-Meier’s survival curves of the overall survival for palbociclib and ribociclib.

As for Kaplan Meier’s survival analysis of PFS, the PFS mean (sd) time for the palbociclib group in months was 17.85 (1.40) 95% confidence interval (CI) [15.11 – 20.59], whereas it was 13.55 (1.66) with a 95% CI of [10.29 – 16.80] for the ribociclib group. The difference between the two groups in terms of PFS was not statistically significant based on the log-rank’s test score (p=0.28), and the Breslow test (p=0.265). Around 50% of the patients had progression-free for 14 months in the palbociclib treatment group, whereas around 50% had the progression-free disease for 11 months in the ribociclib group. The detailed progression-free survival functions in relation to time are indicated in **Table 2**, and the PFS survival curve is illustrated in **Figure 1**. On the other hand, as for the overall survival (OS) of palbociclib and ribociclib, the OS mean time for the palbociclib group in months was 29.82 (1.31) with a 95% CI of [27.26 – 32.39], whereas it was 31.72 (3.65) with a 95% CI of [24.57 – 38.87] for the ribociclib group. The difference between the two groups in terms of OS was not statistically based on either the log-ranks test (p= 0.982), and or the Breslow test (p=0.665). The OS survival functions in relation to time are indicated in **Table 3**, and the OS survival curve is illustrated in **Figure 2**.

**Table 2:** Kaplan-Meier’s survival table of the progression-free survival for palbociclib and ribociclib.

Time in Months	Survival Function	Number of Patients Remaining
Palbocicilb (n=81)		
3	0.975	79
6	0.906	65
9	0.771	51
12	0.572	30
15	0.474	24
18	0.356	16
21	0.303	13
24	0.232	8
27	0.215	6
30	0.198	5
33	0.159	4
36	0.053	0
Ribociclib (n=27)		
3	0.92	25
6	0.815	19
9	0.643	13
12	0.482	7
15	0.347	3
18	0.321	3
20	0.219	2
23	0.000	0

**Table 3:** Kaplan-Meier’s survival table of the overall survival for palbociclib and ribociclib.

Time in Months	Survival Function	Number of Patients Remaining
Palbocicilb (n=81)		
3	0.993	78
6	0.975	68
9	0.956	63
12	0.906	52
15	0.860	44
18	0.829	39
21	0.808	32
24	0.764	23
27	0.665	17
30	0.620	13
33	0.588	11
36	0.442	0
Ribociclib (n=27)		
3	0.980	24
6	0.958	23
9	0.898	18
12	0.838	12
15	0.819	7
18	0.780	5
21	0.751	2
24	0.732	1
27	0.698	1
30	0.657	1
33	0.621	1
36	0.000	0

Furthermore, the results of the cox-regression analysis showed that none of the baseline covariates analyzed in the model was significantly associated with a change in the PFS or in the OS. For the progression-free survival, the overall value of the chi-square test for the model was 5.531 (p=0.938). As for the detailed covariates, none of them was a statistically significant contributor to the PFS ‘age’ (p=0.644), ‘menopausal status’ (p=0.748), ‘the diagnosis of metastasis’ (p=0.246), ‘the type of the metastasis’ (p=0.902), ‘the receiving of prior hormonal therapy’ (p=0.472), ‘the CDK4/6 agent’ (p=0.231), and ‘the CDK4/6 combination medication’ (p=0.548). Similarly, for the overall survival, the overall chi-square test for the model did not show significance (7.389, p= 0.831). As for the detailed covariates, none of them reached the significance level; ‘age’ (p=0.725), ‘menopausal status’ (p=0.756), ‘the diagnosis of metastasis’ (p=0.071), ‘the type of the metastasis’ (p=0.699), ‘the receiving of prior hormonal therapy’ (p=0.990), ‘the CDK4/6 agent’ (p=0.591), and ‘the CDK4/6 combination medication’ (p=0.608).

Regarding the safety outcomes (ADRs outcomes), blood-related side effects and toxicities were the most common among all patients accounting for 73.1% of the population (n=79). Neutropenia was the most common (n=64), followed by thrombocytopenia (n=5), anemia (n=4), febrile neutropenia (n=3), pancytopenia (n=3), and lastly leukopenia (n=1). The blood-related side effects were mostly mild to moderate intensity where grade 1 was present in 49.4% of the patients who developed blood-related side effects (n=39), and grade 2 was present in 34.2% (n=27). As for grade 3 blood-related toxicities were present in 13.9% of the patients, and grade 4 was present in only 1.3% of the patients (n=1). Concerning the gastric side effects, only 7.4% of patients in the total population experienced gastric side effects because of CDK4/6 inhibitors (n=8). Out of those who experienced gastric side effects, diarrhea ± abdominal pain was the most common and was only managed with diarrhea medications such as oral loperamide (n=5). Nausea and vomiting were present only in two patients, whereas constipation was present in only one patient. For the cardiac side effects, QT interval prolongation occurred in 5.6% of the patients (n=6) with an average value of 493 ± 25. Another rare cardiac side effect that occurred only in one patient and led to death was CDK4/6 inhibitor-induced atrial fibrillation. Hepatotoxicity due to CDK4/6 inhibitors occurred in only 1 patient (0.9% of the total population) where the liver enzymes Alanine aminotransferase (ALT) and aspartate aminotransferase (AST) were elevated with values of 255 u/L and 85 u/L respectively. Lastly, for other side effects, 4.6% of the population developed fatigue (n=5), 2.8% developed peripheral neuropathy, 2.8% of the population developed skin rashes of grades 1 and 2, and lastly, 1.9% developed dry eyes syndrome. The side effects in all the populations, specifically palbociclib and ribociclib are summarized in **Table 4**.

**Table 4:** Safety outcomes for the study population (ADRs).

Side Effect	All Patients (N=108)	Palbococlib Group (N=81)	Ribociclib Group (N=27)	p value*
Blood- Related Side Effects, n (%)				
Neutropenia	64 (59.3)	46 (56.8)	18 (66.7)	0.498
Febrile Neutropenia	3 (2.8)	3 (3.7)	0 (0.0)	0.551**
Leukopenia	1 (0.9)	1 (1.2)	0 (0.0)	0.735**
Thrombocytopenia	5 (4.6)	3 (3.7)	2 (7.4)	0.791
Anemia	3 (2.8)	3 (3.7)	0 (0.0)	0.788**
Pancytopenia	3 (2.8)	3 (3.7)	0 (0.0)	0.788**
Cardiac Side Effects, n (%)				
QT-Interval Prolongation	6 (5.5)	1 (1.2)	5 (4.6)	0.004
Induced Atrial Fibrillation	1 (0.9)	1 (1.2)	0 (0.0)	0.735
GI Side effects, n (%)				
Diarrhea	5 (4.6)	4 (4.9)	1 (3.7)	0.792
Constipation	1 (0.9)	1 (1.2)	0 (0.0)	0.735**
Nausea and Vomiting	2 (1.8)	2 (2.4)	0 (0.0)	0.556**
**Hepatotoxicity, n (%)**	1 (0.9)	1 (1.2)	0 (0.0)	0.735**
ALT and AST level, (u/L)	255, 85	255, 85	–	
Other Side Effects, n (%)				
Fatigue	5 (4.6)	4 (4.9)	1 (3.7)	0.792
Peripheral Neuropathy	3 (2.8)	2 (2.4)	1 (3.7)	0.735
Skin Rash	3 (2.8)	2 (2.4)	1 (3.7)	0.735
Dry Eyes Syndrome	2 (1.9)	2 (2.4)	0 (0.0)	0.556**

*The Chi-square statistics with Yates correction at alpha = 0.05; **Fisher exact test at alpha level = 0.05.

## 5 Discussion

CDK4/6 inhibitors are now the mainstay treatment of HR+/HER-2-negative advanced breast cancer in addition to endocrine therapy. In this study, the detailed effectiveness of two of the CDK4/6 inhibiting medications used in the first-line treatment of HR+/HER2- stage IV breast cancer patients in Qatar, palbociclib, and ribociclib, as well as their safety profiles were evaluated. Although the sample size of this study is quite small and the follow-up duration was not too long, it still could successfully provide a valuable comparative insight into these two medications’ effectiveness and safety. With regards to the efficacy, overall, there was no statistically significant difference in the efficacy of the two alternative treatments, palbociclib and ribociclib, with their treatment combinations. In terms of the PFS of the two treatment strategies, the mean PFS in the palbociclib treatment group was 17.85 months, whereas it was 13.55 months in the ribociclib group (p=0.28). Similarly, for the overall survival, the mean survival time for the palbociclib group was 29.82 months, and for the ribociclib, it was 31.72 (p=0.665). Additionally, the safety profiles evaluated the most common blood-related side effects, cardiac toxicities, gastro-intestine (GI) side effects, and hepatotoxicity in the two treatment arms, and the ratio was found to be equivalent between both (p> 0.05). Therefore, the present results would indicate that both medications are equivalent in terms of their efficacy and safety to much extent.

To date, there are no head-to-head randomized controlled trials (RCTs) comparing palbociclib and ribociclib in the treatment of stage IV HR+/HER-2 negative breast cancer population. Only one head-to-head RCT has been currently carried out, but it is yet in the implementation phase and the results have not been published yet (39). Nonetheless, overall, the findings of our study were consistent to much extent with the large population published in phase III trials that compared CDK4/6 inhibitors to the standard care. In an adjusted indirect analysis of the phase III RCTs of CDK4/6 inhibitors, there was no statistically significant difference between palbociclib with its indicated combinations and ribociclib and its indicated combinations in terms of PFS as an indicator of effectiveness. The overall relative risk for palbociclib versus ribociclib according to this analysis in terms of PFS was 0.91 [95% CI (0.75- 1.11)], suggesting that there is no difference between the two treatment strategies (40). In our study, there was also no statistically significant difference between palbociclib and ribociclib with their indicated combinations in the treatment of stage IV HR+/HER-2 negative breast cancer patients.

On the other hand, the observed PFS in our study is overall shorter than the PFS durations published in phase III RCTs. In detail, in a phase III RCT comparing palbociclib plus letrozole to letrozole monotherapy, the median PFS of palbociclib was 24.8 months [95% CI (22.1 to not reached)] versus 14.5 months [95% CI 12.9 to 17.1)] for letrozole group (23). Whereas, in another analysis of an RCT comparing the addition of palbociclib to fulvestrant versus fulvestrant plus a placebo, the median PFS in the palbociclib group was 9.5 months [95% CI (9.2-11.0)] versus 4.6 months [95% CI (3·5-5·6)] in the fulvestrant plus placebo group (24). In our study, the PFS for the palbociclib group with all possible indicated combinations was 17.85 months [95% CI (15.11 – 20.59)]. This may be attributed to our study’s shorter follow-up period compared to the published phase III trials about palbociclib. As for ribociclib, in a phase III RCT that compared the addition of ribociclib to AI versus AI monotherapy in the first-line treatment of HR+/HER-2 stage IV breast cancer women who are postmenopausal, the former comparator had a longer PFS duration of 19.3 months (to not reached during the observational study period) versus 14.7 months in the AI group (20). Additionally, in the MONALEESA-3 trial, ribociclib plus fulvestrant versus fulvestrant monotherapy had a median PFS of 20.5 months (95% CI, 18.5 to 23.5 months) versus 12.8 months in the traditional therapy in the absence of ribociclib (21). In the MONALEESA-7 trial, ribociclib to endocrine therapy versus endocrine monotherapy in premenopausal women, PFS in ribociclib group versus endocrine monotherapy group 23.8 months (95% CI 19.2-not reached) in the ribociclib group compared with 13.0 months (22). However, in our study, the PFS for the ribociclib group was of 13.55 months [95% CI (10.29 – 16.80)]. Similarly, this may be attributed to our study’s shorter duration of patient follow-up.

In terms of overall survival (OS) improvement compared to endocrine monotherapy, ribociclib demonstrated a statistically significant increase for the OS compared to endocirine monotherapy; whereas, palbociclib did not (30–34). However, it is worth noting that the OS periods resulting from this study were also shorter than what was published in the literature. According to a published analysis in 2018 from the PALOMA-3 trial comparing the OS in the palbociclib group plus fulvestrant to the placebo plus fulvestrant as the main outcome, it was 34.9 [95% CI (28.8 – 40.0)] in the palbociclib group, which is longer than what was obtained in our study 29.82 months [95% CI (27.26 – 32.39)] (33). This can also be attributed to the shorter follow-up period in our study as well as the smaller sample size of our study compared to the published studies. For the ribociclib group, a recently published analysis evaluating the OS as the primary outcome in postmenopausal patients receiving ribociclib plus fulvestrant has shown that the addition of ribociclib to fulvestrant was associated with an OS of 66.9% at 42 months with a 95% CI (58.7 to 73.9) (41). In addition, in a recently published abstract for analysis regarding the OS from the MONALEESA-7 trial (in pre/peri-menopausal women receiving ribociclib), the ribociclib treatment was associated with overall survival of median of 58.7 months versus 48.0 months in the placebo group; HR, 0.76 [95% CI (0.61-0.96)] (42). In our analysis, the ribociclib OS was 31.72 months [95% CI (24.57 – 38.87)]. Of note, the follow-up period itself was shorter, which may not reflect the real overall survival. Moreover, the whole population was considered for the survival analysis without a subgroup analysis depending on the menopausal status as per these two previous analyses.

In the present observational study, the two groups were not stratified based on the different menopausal status, treatment combinations, or other baseline characteristics since they were balanced in the baseline characteristics except for the menopausal status. In fact, stratification based on menopausal status was not applicable for several reasons. First, palbociclib is not approved for the treatment of advanced HR+/HER-2 negative breast cancer in premenopausal or perimenopausal status, unlike ribociclib which is FDA-approved for all the menopausal states. Second, till the end of the study follow-up time, for regulatory purposes to save resources at the settings of this study and since palbociclib was already there in the formulary for postmenopausal patients, ribociclib was mainly kept for premenopausal and perimenopausal patients. Therefore, it was almost all pre/perimenopausal patients in the ribociclib arm and postmenopausal patients in the palbociclib arm. Nonetheless, noteworthily, the Cox regression analysis confirmed that this stratification was unnecessary, and the results were valid based on the whole population with different baseline characteristics. According to the research group of this study, there are specifically two clinically significant potentially confounding factors that should have been stratified if found to be proven for significance in previous literature: menopausal status and type of combination therapy. To date, no published head-to-head trials are evaluating the effect of menopausal status factor on the conclusion of the effectiveness of ribociclib (since palbociclib is not indicated for other menopausal states by all means). Nonetheless, ribociclib plus endocrine therapy (tamoxifen, letrozole, or anstrazole) in addition to LHRH agonist (Goserelin) was found to improve PFS in premenopausal patients compared to endocrine monotherapy alone (median PFS of 23.8 months vs 13.0 months respectively) (22). On the other hand, ribociclib plus letrozole resulted in an increased PFS of 25.3 months versus only 16.0 months for letrozole monotherapy in postmenopausal patients (43). As mentioned earlier, there are no head-to-head comparisons between the effect of ribociclib plus endocrine therapy versus endocrine monotherapy in different menopausal states, it can be deduced that the effect is consistent and confirms additive clinical benefit both ways. Concerning the type of combination therapy as another factor, there was one trial presented in the 2023 American Society of Clinical Oncology (ASCO) Annual Meeting comparing the effectiveness of CDK4/6 inhibitors in combination with aromatase inhibitors versus being in combination with fulvestrant; yet, there was no statistically significant difference between both (44). Our study findings based on the COX-regression analysis were consistent with that finding and confirmed that different indicated combinations of medications with CDK4/6 inhibitors have no statistically significant effect on the overall effectiveness of CDK4/6 inhibitors in terms of PFS and OS; and therefore, can be used alternatively depending on the indication and suitability for different patients. The same conclusion could be drawn for the other factors included in this analysis, which included all baseline characteristics.

Lastly, for the safety profile of the two treatments of interest, blood-related side effects: neutropenia, febrile neutropenia, leukopenia, thrombocytopenia, anemia, and pancytopenia were evaluated. Consistently with what was published in the treatments monographs, neutropenia was the most commonly reported side effect for both palbociclib (around 60% of the patients), and ribociclib (66.7%), which was also the most common blood-related side effects for both medications (45, 46). However, febrile neutropenia occurred only in 3.7% of the patients in the palbociclib group. That was followed by thrombocytopenia which occurred in 3.7% of the patients in the palbociclib and 7.4% of the patients in the ribociclib group, which is lower than what was published in the drug monographs (45, 46). This may be because our sample size was smaller than what was conducted in the phase III trials, and so was our follow-up period.

The current study has several strengths to be highlighted. First, it is considered the first retrospective observational comparative study evaluating the efficacy and safety of palbociclib and ribociclib in the real world without the controlled environment of RCTs. To date, only one observational study for palbociclib and ribociclib was carried out; however, it was a retrospective descriptive study, not a comparative one (47). Therefore, the findings of the present study would help researchers and decision decision-makers confirm the findings of the published RCTs in real-world scenarios. In addition, it would help to provide insight not only into the comparative efficacy but also into the safety profiles of the medications. Moreover, it is based on real-world data that was ensured to meet the International Society for Pharmacoeconomics and Outcomes Research and the International Society Pharmacoepidemiology (ISPOR-ISPE) recommendation for good practice related to real-world data use for treatment comparative effectiveness (48).

Nonetheless, similar to any other research, there were some limitations to declare. First of all, the total number of our population in the clinical phase is 108 patients. Although this 108 patient- sample represented the total number of the nationwide population on CDK4/6 inhibitors till the end of the research follow-up period, our sample size was small compared to the other large trials, 333 - 700 patients. Therefore, this may be addressed by future research or a future extension of the current research to include more patients for a longer duration based on power calculations. Secondly, although the follow-up duration was enough for the PFS event to occur, we believe that the follow-up duration was not enough for the OS event, and the reported results are immature. Even in the published phase III trials, the OS results were not mature as they needed a long follow-up duration. Thus, we were forced to report our OS data as they were. Therefore, future research with longer follow-up duration may result in more mature OS data.

The findings of this study can influence the selection and use of palbociclib and ribociclib in stage IV HR+/HER2- breast cancer treatment. Both ribociclib and palbociclib may be equally effective and safe options for patients, so the choice between the two medications should be also based on other considerations such as their cost-effectiveness and availability. Both palbociclib and ribociclib are currently available in Qatar formulary. However, ribociclib was found to be cost-effective compared to palbociclib in the settings of Qatar which may be a factor to consider while selecting between the two medications in the presence of the findings of the current study (49). Further clinical and cost-effectiveness studies should be carried out to include also abemaciclib as the third FDA-approved CDK4/6 inhibitor.

## 6 Conclusions

Since their introduction to the market, the use of CDK4/6 inhibitors is increasing due to their proven clinical efficacy. This research confirmed the clinical benefit of two of the CDK4/6 inhibiting agents, palbociclib, and ribociclib. In addition, we compared them head-to-head for the first time. Our findings showed no statistically significant difference in terms of their PFS or OS. In addition, the distribution of the ADRs between the two treatments was balanced, suggesting that the two treatments have similar safety profiles. Other factors that may be thought to affect the effectiveness of the two medications were also evaluated. We proved that these factors, such as the type of combination medication, have no significant effect on the effectiveness of the two CDK4/6 inhibiting medications. By providing rigorous and reliable data on the safety and effectiveness of ribociclib and palbociclib, this research contributes to a better understanding of the options available for breast cancer treatment and helps to guide clinical decision-making in this important area. Since this research was associated with the limitations of small sample size and short follow-up durations, further research to address the study limitations, such as larger-scale studies, should be considered in the future.

## Data availability statement

The original contributions presented in the study are included in the article/supplementary material. Further inquiries can be directed to the corresponding author.

## Ethics statement

The studies involving humans were approved by The study was firstly ethically approved by the Medical Research Center (MRC) on January 30, 2020, under the protocol approval number: MRC- 01-19-318, followed by the approval from the Qatar University International Review Board (QU-IRB) on February 10, 2020, under the approval number: QU-IRB- 1231- E/20. The studies were conducted in accordance with the local legislation and institutional requirements. Written informed consent for participation was not required from the participants or the participants’ legal guardians/next of kin in accordance with the national legislation and institutional requirements.

## Author contributions

All authors contributed to the work and approved the final version of the manuscript.
